# Comparison of Three Macroinvertebrate Sampling Methods for Use in Assessment of Water Quality Changes in Flashy Urban Streams

**DOI:** 10.4236/jep.2020.118035

**Published:** 2020-08-05

**Authors:** Roger Yeardley, Scott Jacobs, Ken Fritz, William Thoeny

**Affiliations:** 1 Office of Research and Development, U.S. Environmental Protection Agency, Cincinnati, USA; 2 Pegasus Technical Services, c/o U.S. Environmental Protection Agency, Cincinnati, USA

**Keywords:** Macroinvertebrate, Methods, Stream, Urban, Sampling, Flow, Hydrology

## Abstract

The unique challenges associated with sampling of macroinvertebrates in flashy urban streams create a methods gap. These streams form isolated pools for much of the year, interspersed with spates that scour and deposit excessive amounts of sediment. Commonly used stream grab sampling methods, such as nets and Hess and Surber fixed-area samplers, work well in wadable streams with perennial flow. Deployed samplers (Hester-Dendy, gravel tray) can be used in waters with or without flow. We evaluated three methods which don’t require stream flow: modified Hester-Dendy (MHD), gravel tray, and bucket (a type of cylinder grab sample method), for their potential use in bioassessment of a project involving daylighting of a 180-m culvert on Congress Run, a flashy urban tributary to Mill Creek in Cincinnati, Ohio. Method efficacy was measured using three criteria: usability (level of effort and recoverability of samplers), variability, and community retrieval completeness. The bucket method required the lowest level of effort and had the highest sample recovery. The bucket sampler had the lowest variability for most metrics, including the critical metric of taxa richness, with a coefficient of variation (CV) of 20.9%. The MHD and tray samplers had taxa richness CVs of 42.9% and 53.9%, respectively. The bucket sampler also had the lowest CV (27.4%) for a multi-metric index. The bucket sampler performed best with respect to community retrieval completeness, with higher pooled and average taxa richness. The total number of taxa collected from all the replicate bucket grab samples (42) was greater than that collected by the HD and tray samplers combined (27). Multivariate analyses showed significant grouping with respect to methods and location. This study supports the bucket grab sampler method as a candidate for sampling of flashy urban streams.

## 1. Introduction

Bioassessment through sampling of macroinvertebrate communities is a well-established means of measuring the effects of multiple stressors on stream water quality. Changes in macroinvertebrate communities can be used to assess the success of stream restoration and Green Infrastructure (GI) projects. For the widely used means of sampling macroinvertebrates in streams, flow is either required or highly recommended. The flow regime of flashy urban streams results in a series of interspersed and poorly connected pools at baseflow alternating with brief but destructive flooding events. These physical/hydrological conditions pose challenges to common sampling methods. Therefore, none of the popular sampling methods are ideal for bioassessment of flashy streams.

There are a significant number of streams suffering from the effects of urbanization, including flashy flow from high percentages of impervious surfaces. In 2000, the U.S. EPA [1] estimated that over 130,000 km of streams in the U.S. were impaired by urbanization. This number has likely increased significantly in the two decades since that report. Many of these urban streams would be expected to be intermittent or ephemeral and consist of a series of pools for much of the year, and to not have the requisite flow to effectively use these popular methods. This indicates a significant methods gap regarding an effective macroinvertebrate sampling method for sampling flashy urban streams.

Stressors in urban streams include contaminant input, which can come from sources such as sanitary sewer overflows (SSOs), combined sewer overflows (CSOs), industrial discharges, septic systems, wastewater treatment plant effluents, irrigation, and roadway runoff. However, there is evidence that the biotic integrity of streams, as measured by their macroinvertebrate communities, can be determined as much by alterations in hydrology and stream flow regimes [2] [3] as by contaminant input. For instance, Hawley *et al*. [4] found that a reference stream went from a biotic integrity index rating of “excellent” to a rating of “poor” (and saw similar decreases in metrics like taxa richness) in a year with an unusually high frequency of critical discharge events, which mobilized the stream bed enough to significantly disturb the benthic macroinvertebrates. Flashy flow regimes, with relatively high peak flows and low base flows [3] [5] [6] are caused or exacerbated by the high percentage of impervious surfaces in urban drainage basins. This flashiness, part of an “urban stream syndrome” [6], can result in streambed scouring and sediment mobilization episodes combined with low to no flow during much of the year.

There are a number of macroinvertebrate sampling methods historically used to sample streams. Nets (e.g., D-frame, kick net) are generally considered a qualitative to semi-quantitative method and are the most widely used method by government environmental agencies in the U.S. [7] [8] [9] [10] [11] and around the world [12]. For these methods, macroinvertebrates dislodged from the substrate are carried by flowing stream water into the nets, and thus these methods largely require stream flow. Fixed-area grab samplers (e.g., Surber, Hess, bucket/cylinder) are quantitative methods that are widely used [7] [9] [10] [12] [13] and give a “snapshot” of stream conditions. Ohio EPA [11] uses Surber samplers in their assessment of headwater streams. Surber and Hess fixed-area samplers also take advantage of stream flow to wash invertebrates into nets positioned downstream of the sampling area.

Deployed artificial substrate samplers, which are deployed in a water body at one point in time and retrieved and processed later (usually a period of weeks), can give an integrated picture of water quality. Two of the most common types of deployed artificial substrate samplers are Hester-Dendy (HD) multi-plate samplers and rock baskets or gravel trays [7] [14]. Hester-Dendy multi-plate artificial substrate samplers are used by the U.S. EPA, USGS, and several state environmental agencies [7] [9] including Ohio EPA [15] [16] [17]. The Ohio EPA [15] [16] [17] recommends a flow of ≥10 cm/sec for use of Hester-Dendys in Ohio streams. The Iowa Department of Natural Resources [18] recommends a flow of approximately 15 – 50 cm/sec for their standard Hester-Dendys. IDNR has a special low-flow modified Hester Dendy, but some flow is recommended for use of this as well. Though some government agencies recommend a certain level of flow for use of HDs and gravel trays in streams and other lentic habitats, these artificial substrates can provide habitat for macroinvertebrates to colonize, regardless of flow presence and thus their use does not require flow. Another method which does not require stream flow, a cylinder fixed-area sampling method using a bucket [13], would also seem to be a candidate for sampling the pools that exist for much of the year in flashy urban streams.

**Figure 1.**
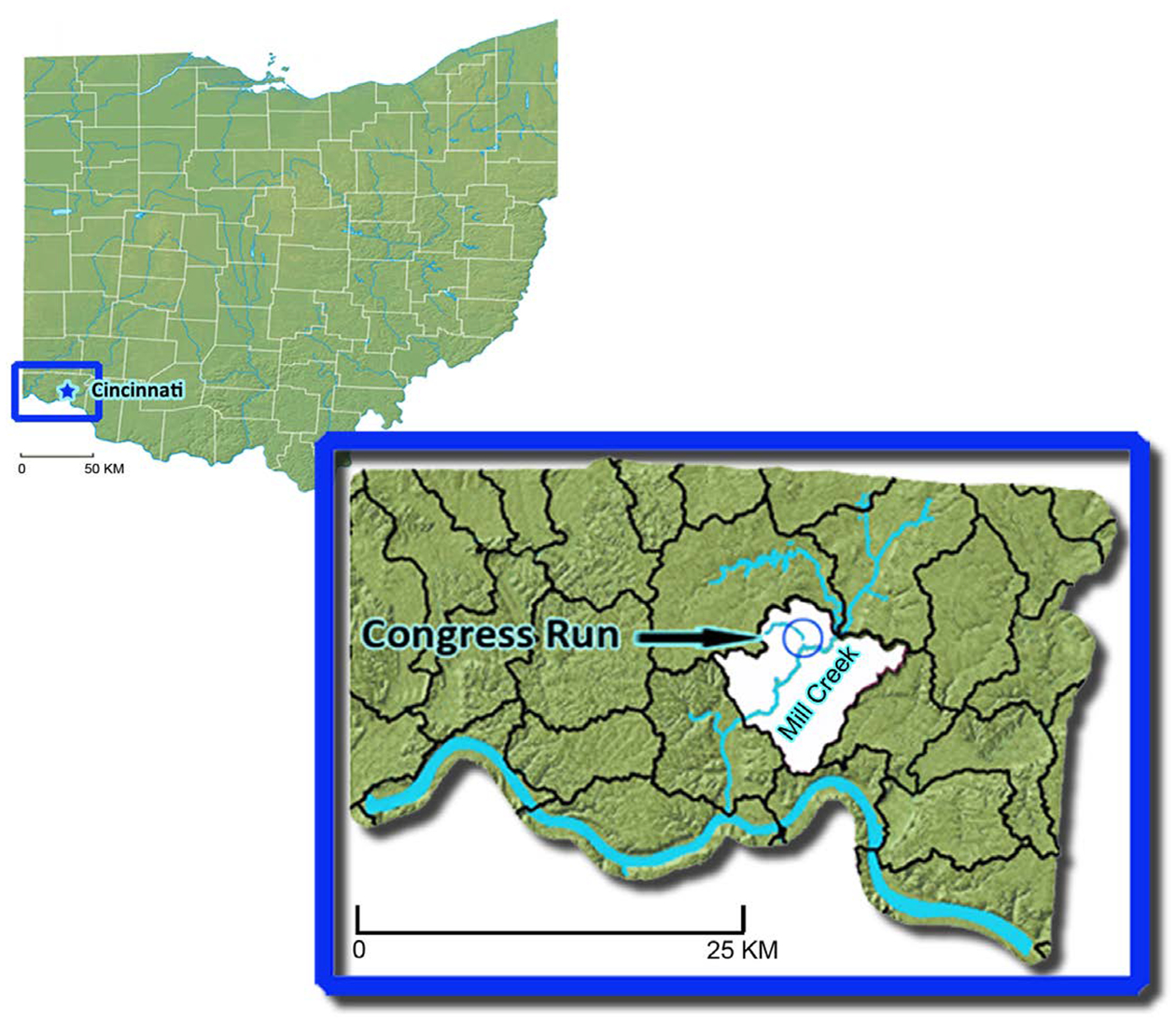
Study area—Congress Run, tributary to the Mill Creek in Cincinnati, Ohio near its confluence with Mill Creek. Hydrologic unit in which Congress is situated (HUC-12 # 050902030105) highlighted in white. Insert is Hamilton County, in which Cincinnati is located.

Bioassessment of benthic macroinvertebrate communities was chosen as one element of a research project assessing the effectiveness of a GI project daylighting a culvert on Congress Run stream, in the Congress Run-Mill Creek hydrological unit code (HUC) #050902030105 (Figure 1) in Cincinnati, Ohio. The Congress Run HUC drains 77.6 km^2^ and has the significant amount of impervious surfaces that would be expected to accompany 79% developed land [19] [20]. GI technologies, such as rain gardens, green roofs, permeable pavements, and daylighting, encourage a more natural flow of water. Daylighting projects, like that at Congress Run, involve restoration of stream sections that have been channelized and diverted through pipes. Daylighting and related efforts help to reverse channelization and concentration of flow in streams and rivers caused by culverts, pipes, and concrete lining of streams. Other GI technologies encourage more infiltration of water from precipitation (with more immediate storage and groundwater recharge) through plants and soils, and more evapotranspiration, as opposed to direct runoff from impervious surfaces of roads and urban structures.

To maximize the ability to detect differences in communities before and after remediation or GI projects or in different reaches, a macroinvertebrate sampling method is desired that is usable in a stream with flashy flow patterns, has a relatively low variability, and achieves as complete as possible retrieval of the taxa present in that stream reach. We compared the efficacy in assessing pool habitat in a flashy urban stream of three methods which don’t require steam flow: bucket grab sample, gravel tray, and modified Hester-Dendy (MHD), with respect to these criteria. The method that rated the best with respect to these criteria would then be used for continued bioassessment before and after daylighting to help evaluate whether this GI project had resulted in improvement of stream condition.

## 2. Materials and Methods

### 2.1. Physical Measurements

Stream flow was measured (Swoffer Model 3000) in the middle of the water column (3 replicate measurements) near the upstream entrance to each pool where the flow would be expected to be greatest and therefore most detectable in these low flow conditions. Sediment size composition and organic content were characterized to assess whether differences in sediment characteristics among methods affect the macroinvertebrate communities recovered by each method. For the MHD and tray samples, the sediment characterized represented that which was deposited on them during the deployment period. Sediment particle size fractions were analyzed by wet and dry sieving of the sediments collected by bucket, tray, and HD samples after the sediment samples were sorted and macroinvertebrates removed. Contents of each jar containing the sorted sample were rinsed onto a 2 mm sieve stacked on top of a 250 μm sieve. Sediment on the 2 mm sieve was swirled and rinsed until particles < 2 mm were passed through the sieve and collected on the 250 μm sieve. Contents of the sieves were transferred to pre-ashed aluminum pans, air-dried, then oven-dried for 24 hours at 50°C, followed by ashing in a muffle furnace for 3 hours at 550°C. Ash-free dry mass (AFDM) procedures based on ASTM methods [21] [22] were used to measure the total dry weight of sediment, the organic and inorganic sediment weights and percentages of two sediment size fractions—250 μm to 2 mm (fine particulate organic matter (FPOM) for the organic portion), and >2 mm (coarse particulate organic matter (CPOM) for the organic portion). As a rough measure of larger sediment particles, medium to coarse gravel and larger [23] [24], the >2 mm fraction was dry sieved in a 5.6 mm sieve and the weight of this larger size fraction also recorded. These analyses do not account for fines and silt as these were lost in the initial sieving of sediments at 250 μm to recover macroinvertebrates.

### 2.2. Samplers

#### 2.2.1. Gravel Trays

**Figure 2.**
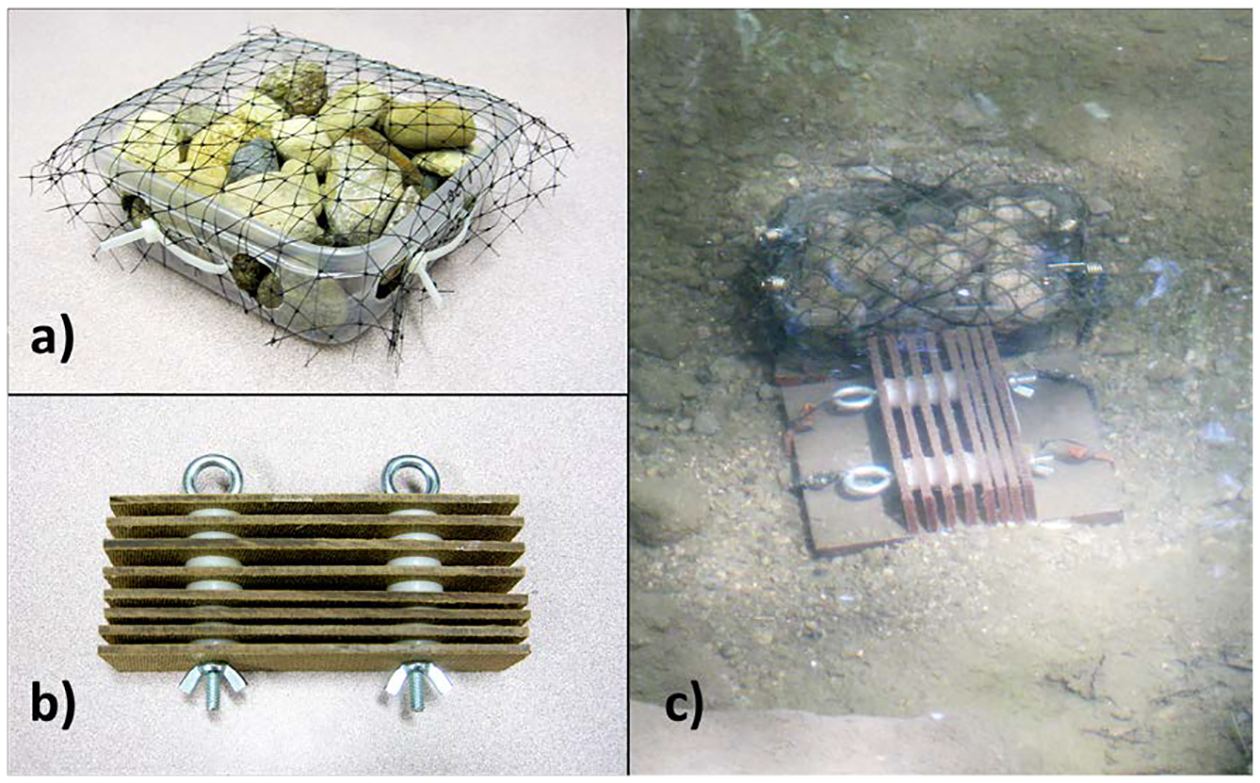
Deployed samplers: tray and low-profile Hester-Dendy. (a) Assembled gravel-filled tray; (b) Assembled modified Hester-Dendy (MHD); (c) Gravel tray and Hester-Dendy deployed in a pool on Congress Run.

The trays used to hold the gravel were plastic Ziploc™ Small Square containers (1.5 Pt./709 ml). One-inch holes were drilled in the bottom and sides to allow connectivity with the stream water and sediment; four holes on the bottom and two on each side. Between 1300 and 1400 g of Vigoro™ brand natural river rock, ranging in diameter from 1.5 to 5 cm, was loaded into each tray. Depending on the classification scheme, this size range of rocks would be classified as a mix of gravel and pebbles [23] [25] or as gravel [24] [26] [27]. Gravel volume and interstitial space were measured by a liquid displacement method. After weight and volume of gravel were measured, each filled tray was covered with two layers of 1.9 cm square-opening cotton/nylon mesh/fishing net to prevent stream flow from dislodging gravel during deployment (Figure 2(a) and Figure 2(c)). Mesh was fastened to the trays with zip ties. These samplers had an average of around 200 ml of interstitial space for macroinvertebrates to attach and occupy, and a cross-sectional area/ footprint of 195 cm^2^.

#### 2.2.2. Modified Hester-Dendys (MHDs)

The design of the multi-plate samplers [28] used in this study was a low-profile modification of versions used in previous studies [14] [15] [16] [17]. Each sampler was made up of eight rectangular Masonite/hardboard plates (3.8 × 15.2 cm each) separated by nylon washers (3 mm thick, 8 mm Hole Size, 8 mm ID, 22 mm OD) and held together by two eyebolts (Figure 2(b) and Figure 2(c)). There were 2 widths of spaces in each HD, 3 spaces between plates created by single spacers, and 3 spaces created by double spacers. Each of these samplers offers the same area for colonization as an Ohio EPA style HD. Our MHDs offer the same area per plate (58.1 cm^2^) as the Ohio EPA’s 76.2 × 76.2 cm (3 × 3”) plates, and also have 8 plates per HD. These “low profile” MHDs were designed to stay submerged in pools and riffles of headwater streams, which are often shallow, and to provide less resistance to flow during times of high discharge. Each sampler has 158 ml of interstitial space for macroinvertebrates to attach and occupy, and a cross-sectional area/footprint of 96.8 cm^2^.

#### 2.2.3. Bucket Grab Samplers

**Figure 3.**
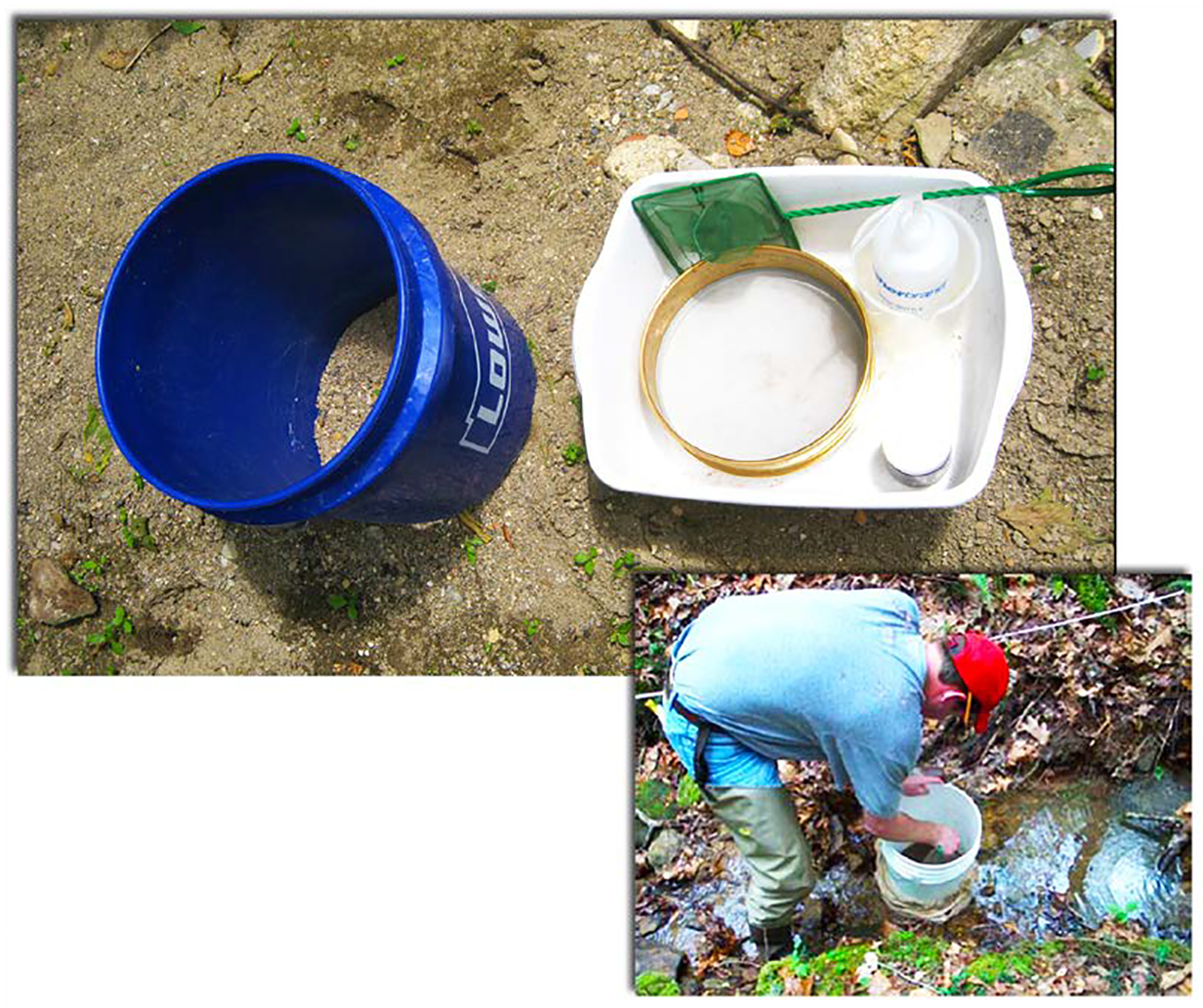
Bucket grab sampling method, showing equipment and sampling being performed.

The bucket grab sampling method was based on that described by Fritz *et al*. [13]. The equipment included an 18.9 L (5 gallon) bucket (Figure 3), with the bottom removed and the bottom edge modified to be serrated. This type of sampler works best in areas without concentrations of larger substrate such as cobble and boulders, and with some combination of silt, sand, and fine through medium gravel into which the bucket can be pushed. Other important equipment included a small hand net (~250-μm mesh), a small plastic tub, 250 μm sieve, squeeze bottle(s), and sample containers and ethanol for stream side preservation of the sample. The 25.4 cm (10 in.) diameter bucket delineates a 506.7 cm^2^ sampling area.

### 2.3. Sampling Process

**Figure 4.**
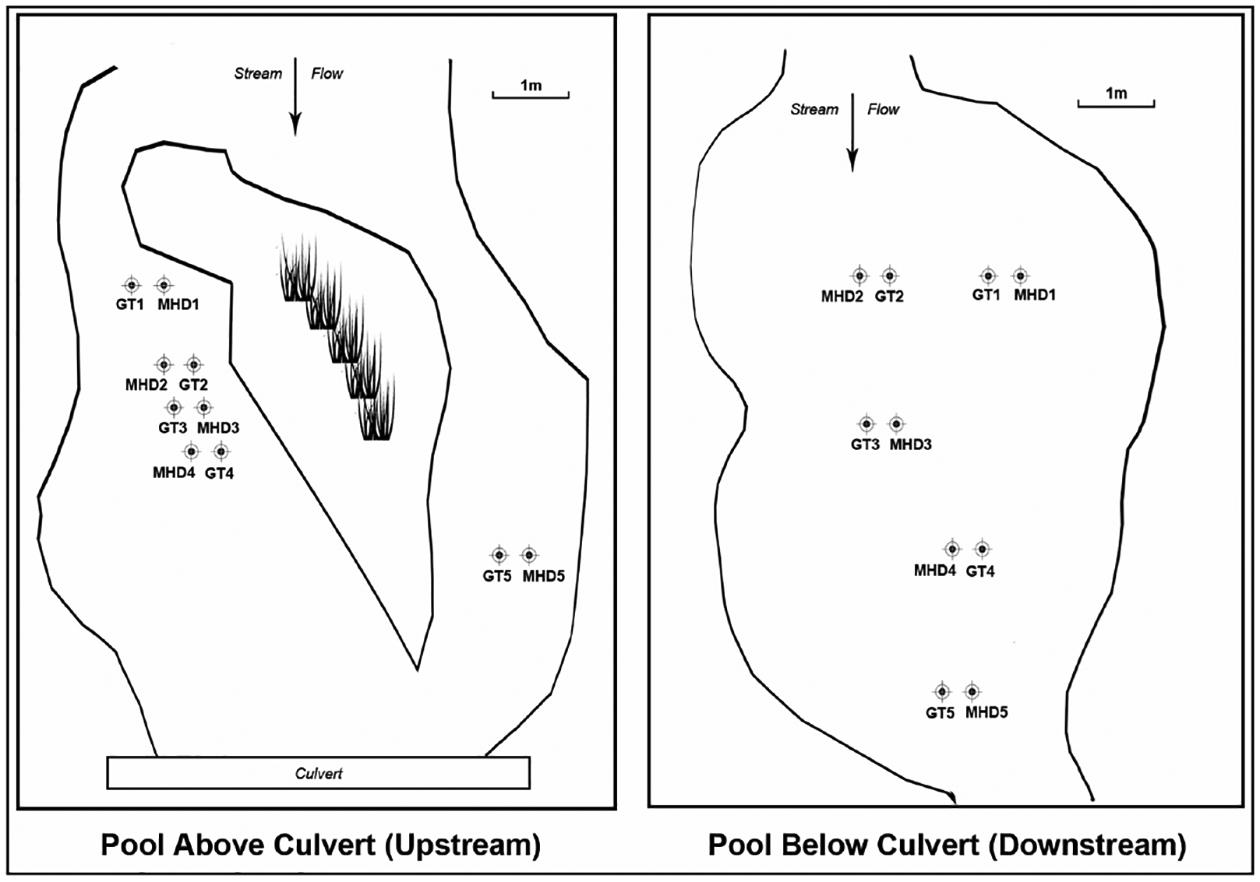
Illustration of the locations of the MHD and tray samplers in the upstream and downstream pools.

Five pairs of MHD and gravel tray samplers were deployed in the deeper parts of pools upstream and downstream from the culvert. The locations of the samplers within these pools are shown in Figure 4. The MHD samplers were situated with the plates perpendicular to stream flow. At the stream site, to better anchor them, MHD and gravel tray samplers were attached to paving stones or steel plates (15.2 × 15.2 × 1.9 cm) with elastic cords (Figure 2(c)). The samplers were deployed in the stream for 6 weeks. After 6 weeks deployed samplers and grab samples were collected (in a downstream to upstream order) over a five-day period in June 2017. No significant rain events occurred during this five-day period (Figure 6). When the buried trays and MHDs were dug up, the several centimeters of sediment on top was not part of the sample analyzed for macroinvertebrates, only the sediment in the samplers themselves. Bucket grab samples were collected in the pools near the locations, along the same transects as the MHD and tray samplers on the same day or within one day of retrieval of these deployed samplers. Water depth at each sampler was measured at the start and end of the deployment. MHD and tray samples were put into resealable plastic bags and kept on ice in a cooler until processed at the lab. Sample processing began within 3 hours of retrieval of the first sample. All samples were sieved with USA. Standard No. 60 sieves (250 μm) and preserved on the day of collection in 80% ethanol.

Bucket, or “stovepipe” samplers have been shown useful in shallow aquatic habitats with little or no flow [13] [29] [30] [31]. Per Fritz *et al*. [13], for our bucket grab samples, a bucket with the bottom removed and serrated edges, was screwed into the sediment. The top ~10 cm of sediment was stirred by hand, and then immediately a hand net was passed through the water column for ten seconds to collect macroinvertebrates and sediment. This stirring and netting process within the sample area was repeated three times. Contents of the net were placed into a 250 μm sieve. The sediments in these pools where grab samples were taken consisted mostly of sand, silt, and fine gravel. The net contents from grab samples were also augmented with the scrapings from 3 – 4 rocks in the pool. Samples were placed in jars and preserved in 80% ethanol.

### 2.4. Macroinvertebrate Identification

Prior to sorting, samples were split in half. If a sort of half the sample resulted in a count of less than 100, then the other half was sorted as well, for a total count. Though it would have been preferable to do a subsample count of 300 – 500 individuals as many state agencies do [8], this was not possible due to low abundance in most of the samples from deployed samplers. However, the lower subsample count of 100 is not uncommon among state agencies [7]. For samples where counts are derived from a sort of half of the sample, the other half received a qualitative sorting for any taxa not found in the initial half. If found, these additional taxa were included in taxa richness measures. Macroinvertebrates were identified to genus or species using keys by Merritt *et al*. [32], Epler [33], and Simpson and Bode [34]. Chironomid specimens were subsampled for genus- or species-level identification proportionally to the relative abundance across chironomid tribes.

### 2.5. Statistical Analysis

One-way Analysis of Variance (ANOVA) tests were run in SigmaPlot™ 14 to compare taxa richness and abundance between methods. Data that passed the Shapiro-Wilk normality test were analyzed using the Holm-Sidak multiple comparison procedure. Data that did not pass the normality test were analyzed by the nonparametric Kruskal-Wallis test. The Invertebrate Community Index (ICI), which is the Ohio EPA’s principle measure of overall macroinvertebrate community condition [17] and consists of 10 structural community metrics, was calculated for each sample.

Nonmetric Multidimensional Scaling (NMDS) ordinations and Multi-Response Permutation Procedure (MRPP) analyses [35] were conducted and graphs were generated using PC-ORD™ software [36]. A Sorenson (Bray-Curtis) distance measure was used in the NMDS analyses. MRPP was used to test whether there were significant differences among methods or between locations. MRPP analyses were run using a Relative Sorenson distance measure and the distance measure was rank transformed. The test statistic, T, describes the separation between groups by comparing an observed delta to an expected delta. The more negative T is, the stronger the separation [35] of groups. The observed delta represents the average within-group distance. The calculated expected delta represents the mean delta for all possible partitions of the data. The agreement statistic, A, is the chance-corrected within-group agreement or homogeneity. An A of 1.0 would indicate that all taxa are identical within groups. In practice, values for A are commonly below 0.1; A > 0.3 is considered fairly high [35]. The p value is used to evaluate how likely an observed difference is due to chance, i.e., the like-lihood of getting a delta (the average within-group distance) as small, or smaller than the observed delta.

## 3. Results

Water depths above the deployed samplers ranged from 18 to 50 cm (avg. = 35 cm, SD = 10) upon deployment and from 3 to 35 cm (avg. = 20 cm, SD = 11.5) upon retrieval, with the pool just upstream of the culvert being slightly shallower than the more permanent pool downstream of the culvert. There was a slight flow upon deployment; averaging 0.047 m/s above the culvert and 0.020 m/s downstream of the culvert, and no measurable flow at either site upon retrieval.

**Figure 5.**
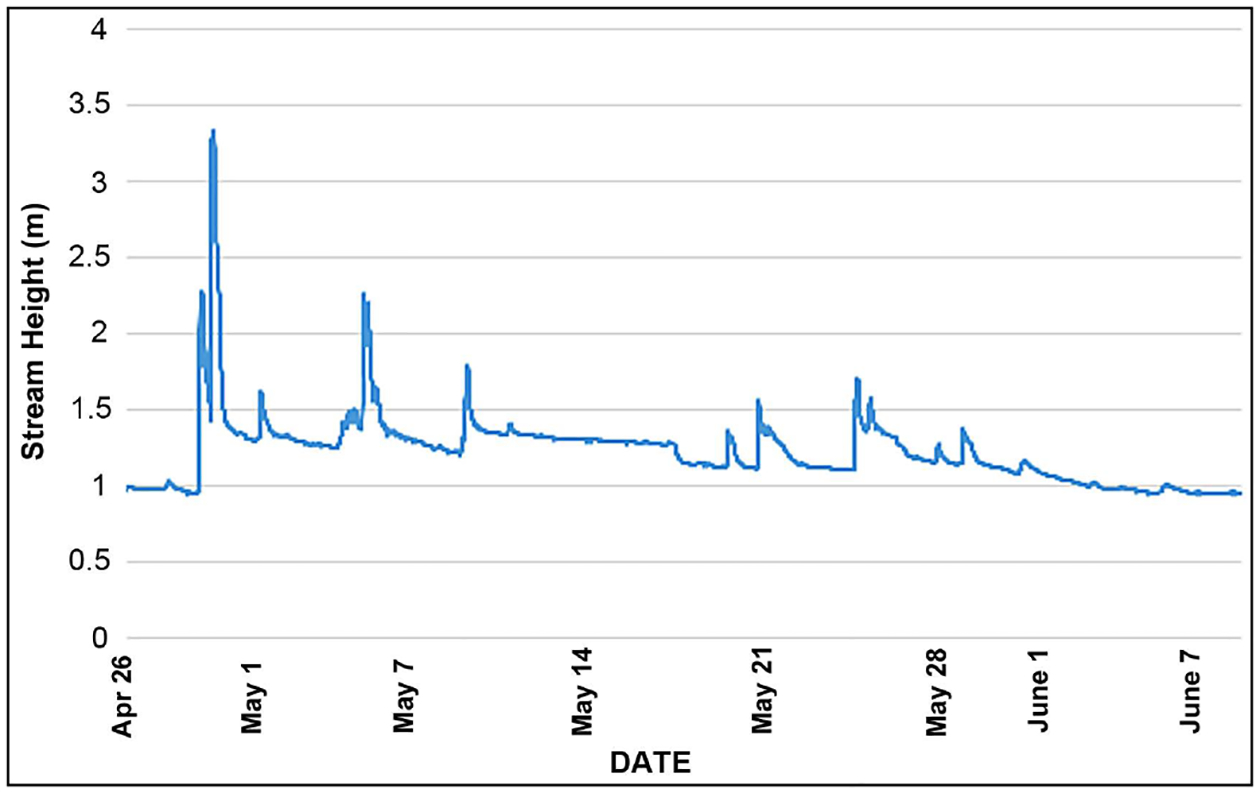
Stream stage measurements for the period of deployment of Hester-Dendy and gravel tray samplers, showing the surges in stream height caused by rain events. Measurements are from USGS stream gage 03259198 (Congress Run near Carthage OH).

All 5 trays and 4 of 5 MHDs were retrieved from the pool above the culvert, though many of these were buried by sediment. From the pool below the culvert, only 2 of the 5 pairs of deployed samplers (2 MHDs and 2 trays) were retrieved and these were also buried by sediment (though not as deeply buried as the upstream samplers). It is believed that the samplers that could not be found below the culvert were washed away, possibly due to the pipe that makes up the culvert concentrating and speeding up the flow when it is filled to capacity in large rain events. Congress Run experienced a quite large surge due to a rain event just a few days after MHDs and trays were deployed (April 26, 2017). Starting in the early morning of April 28, in a matter of about 12 hours the stream rose over 2.2 m from a base flow of just a trickle (Figure 5), to overflowing its banks. The base flow of approximately 1m in this graph does not represent 1m depth of water, but a minimal base flow. The graph of stream stage (height) during the deployment period in Figure 5 demonstrates the flashy nature of this urban stream, showing this and other smaller but significant rain events. Stage of water is used in the graph, as discharge was not yet available from the U.S. Geological Survey (USGS) for this gage. The extent to which samplers were buried and the significant amounts of sediment that accumulated, especially in the gravel trays, are also testament to the large amounts of sediment deposited with the flashy flows that accompanied each significant rain event. Though the heavy rain event and associated surge in stream height and discharge that occurred within a few days of deployment of MHDs and trays (first 2 peaks in Figure 5) were quite large, this kind of surge is not unusual in this watershed.

There was a marginally insignificant difference (p = 0.052) in percent organic content of the sediments among the methods. The tray method recovered a significantly greater dry mass of sediment (median = 496 gm, p < 0.001) than either the MHD (median = 172 gm) or bucket method (median = 59 gm). By sieving sediment after sorting out the macroinvertebrates, we were able to do a general examination of some particle size classes [23]; sand (250 μm - 2 mm), fine to medium gravel (2 mm – 5.6 mm), and medium to coarse gravel (>5.6 mm). The bucket and tray methods agreed well with respect to the fraction of sand and larger particles that they collected, with the makeup of sediment deposited for each one being about 35% sand (250 μm to 2 mm) and around 60% gravel (>2 mm). These two methods also agreed in that 77% of the gravel fraction was medium to coarse gravel (>5.6 mm), with a visual inspection showing the majority of these rocks being >10 mm, many greater than 20 mm. By comparison, the MHDs collected 49% sand (250 μm to 2 mm), 48% gravel (>2 mm), with less of the gravel fraction (~30%) being medium to coarse gravel (>5.6 mm). The tray samples had the greatest dry weight of sediment collected in them (avg. = 278 gm), followed by the MHD samples (avg. = 164 gm) and bucket grab samples (avg. = 107 gm).

### 3.1. Usability

We were able to easily sample 1 – 2 sites per day (with 5 replicate samplers per site) with one person using the bucket method. With sample handling, transport to the lab and sample processing at the lab (sieving, sampler disassembly and scraping (MHDs)), it took two people a full day to complete one site (with both trays and MHDs). There were also differences seen in sample sorting time, with tray samples taking over 7 hours per sample to sort, and bucket and MHD samples taking approximately 4.5 hours per sample. For all but one of the deployed samplers, the whole sample was sorted for a total count of organisms present. For the bucket grab samples, which had far greater total numbers, total counts were done for only two of the ten samples. For the other 8 samples, one-half of the sample was sorted and identified.

### 3.2. Variability

The bucket grab method had the lowest coefficient of variation (CV) for a number of common taxa richness and abundance metrics, as well as for the ICI multi-metric index of water quality (Table 1). The bucket grab method also had the lowest CV for number of dipteran taxa, and Chironomini, Tanytarsini, and Tanypodinae abundance. For Oligochaete (Table 1) and Orthocladiinae abundance, the CVs for the bucket grab method were the highest of the three methods.

### 3.3. Macroinvertebrate Community Retrieval Completeness

Table 2 shows the taxa that were collected in this reach of Congress Run. The majority (30 of 43) of taxa collected were of the order Diptera. For this spring sampling, no individuals from the Ephemeroptera, Plecoptera, and Trichoptera (EPT) taxa were found in any of the samples.

**Figure 6.**
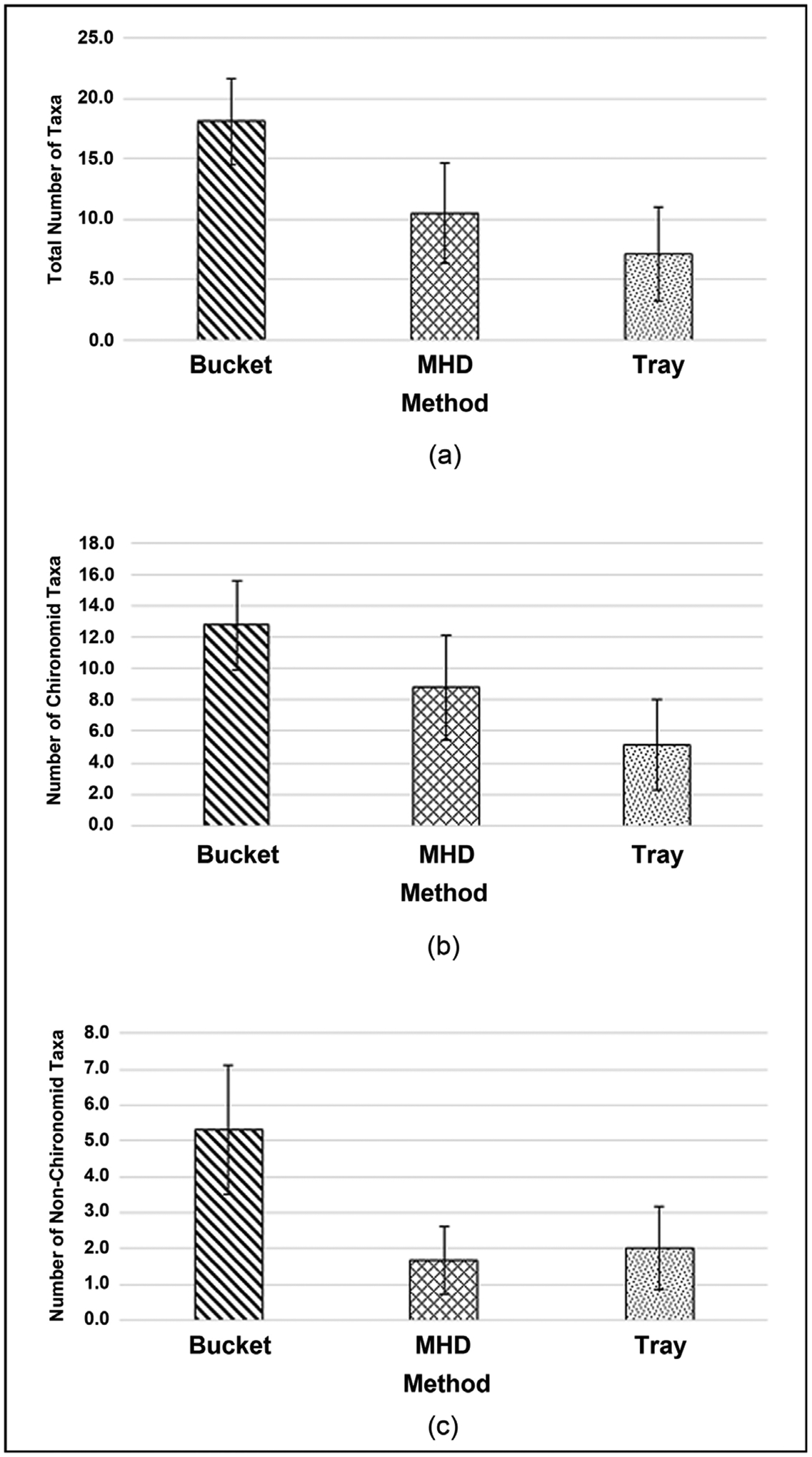
Macroinvertebrate taxa richness metrics by sampling method (a) mean total taxa richness ± 1 SD; (b) mean chironomid taxa richness ± 1 SD; (c) mean non-chironomid taxa richness ± 1 SD.

ANOVAs showed some differences in taxa richness among methods. The bucket grab sample method produced a greater taxa richness per sample (Figure 6(a)) than the MHD (p = 0.003) and tray (p < 0.001) methods. No difference was seen in taxa richness between the MHD and tray (p = 0.147) methods. The bucket grab method collected a significantly higher number of chironomid taxa per sample (Figure 6(b)) than the tray (p < 0.001) and MHD (p = 0.047) methods. There was a significant difference in number of chironomid taxa between the MHD and tray methods (p = 0.047). The bucket grab method collected more non-chironomid taxa per sample (Figure 6(c)) than the MHD (Kruskal-Wallis; p = 0.003) and tray (p = 0.007) methods. No difference was seen in number of non-chironomid taxa between the MHD and tray (p = 1.000) methods.

**Figure 7.**
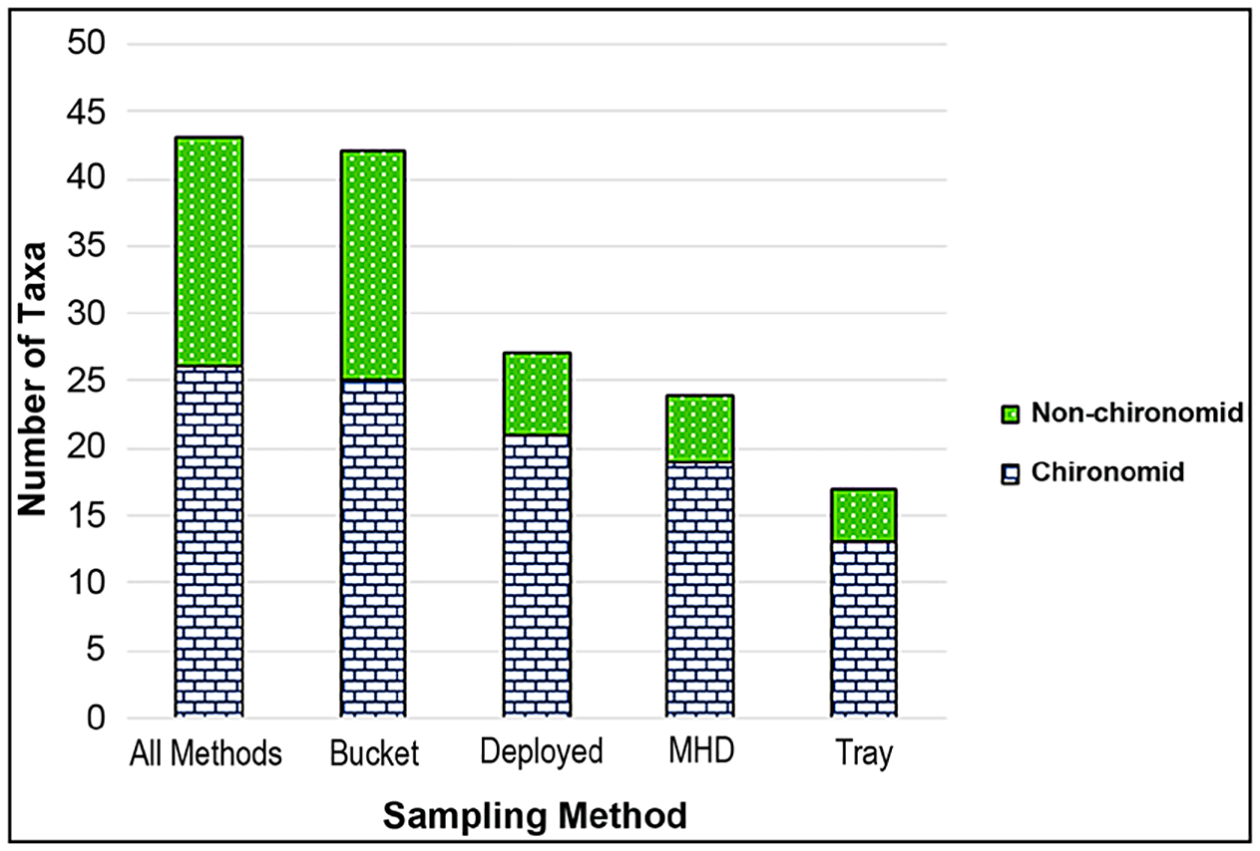
Total number of taxa collected from all the replicates of each method and combinations of methods (all deployed samplers (MHD + Tray) and all methods).

In addition to analysis of the mean of taxa richness per sample, we calculated the total taxa collected from all the replicate samples of the methods (Figure 7). The number of taxa retrieved from all the bucket grab samples (42) was very close to the number of taxa retrieved from all samples, all methods (43). The MHDs retrieved 24 taxa and the trays retrieved 17 total taxa. There were more bucket grab samples collected (10) than MHD (6) or tray (7). However, the combined total taxa collected from of all 13 deployed samplers (MHD + tray), was also lower (27) than the total collected from 10 bucket grab samples (42).

**Figure 8.**
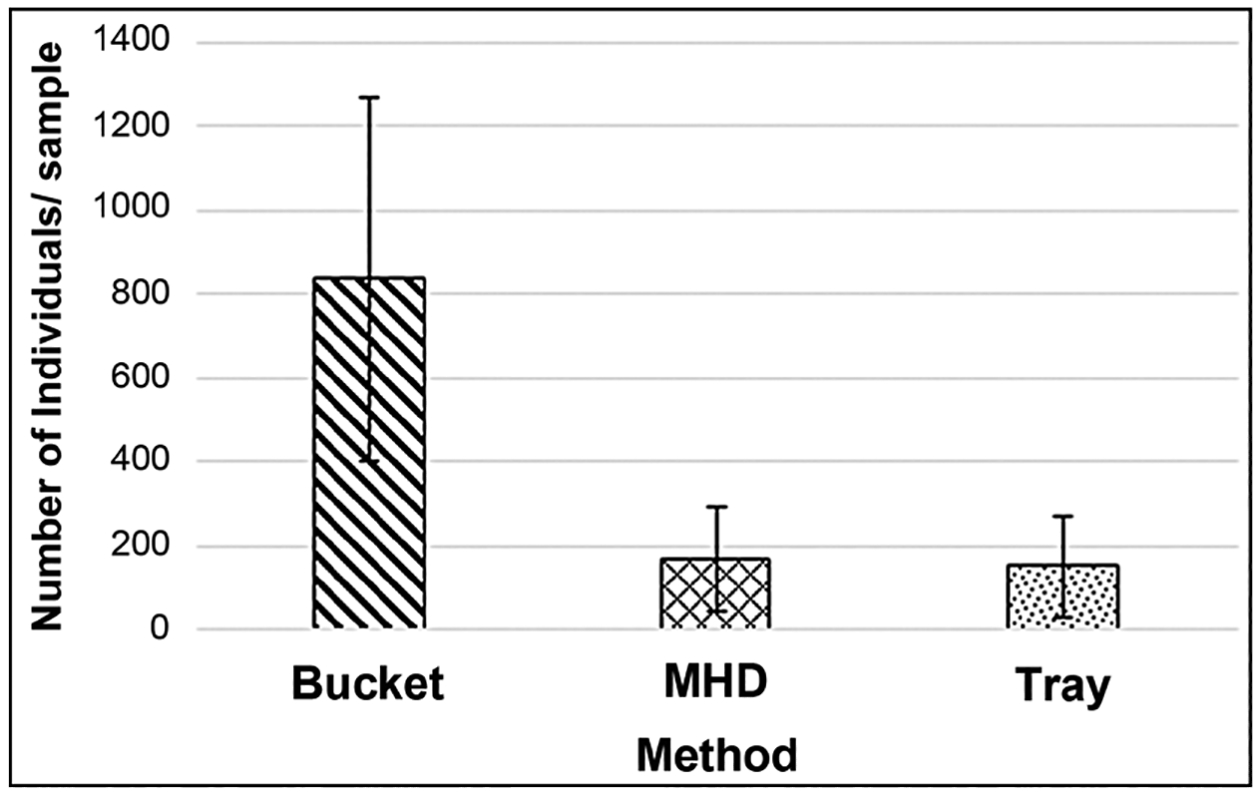
Comparison of mean abundances ± 1 SD collected by the 3 methods.

The Bucket method provided a significantly higher abundance per sample (Figure 8) than the MHD (p = 0.007) and tray (p = 0.005) methods. No difference was seen in abundance between MHD and tray (p = 1.000) samplers.

**Figure 9.**
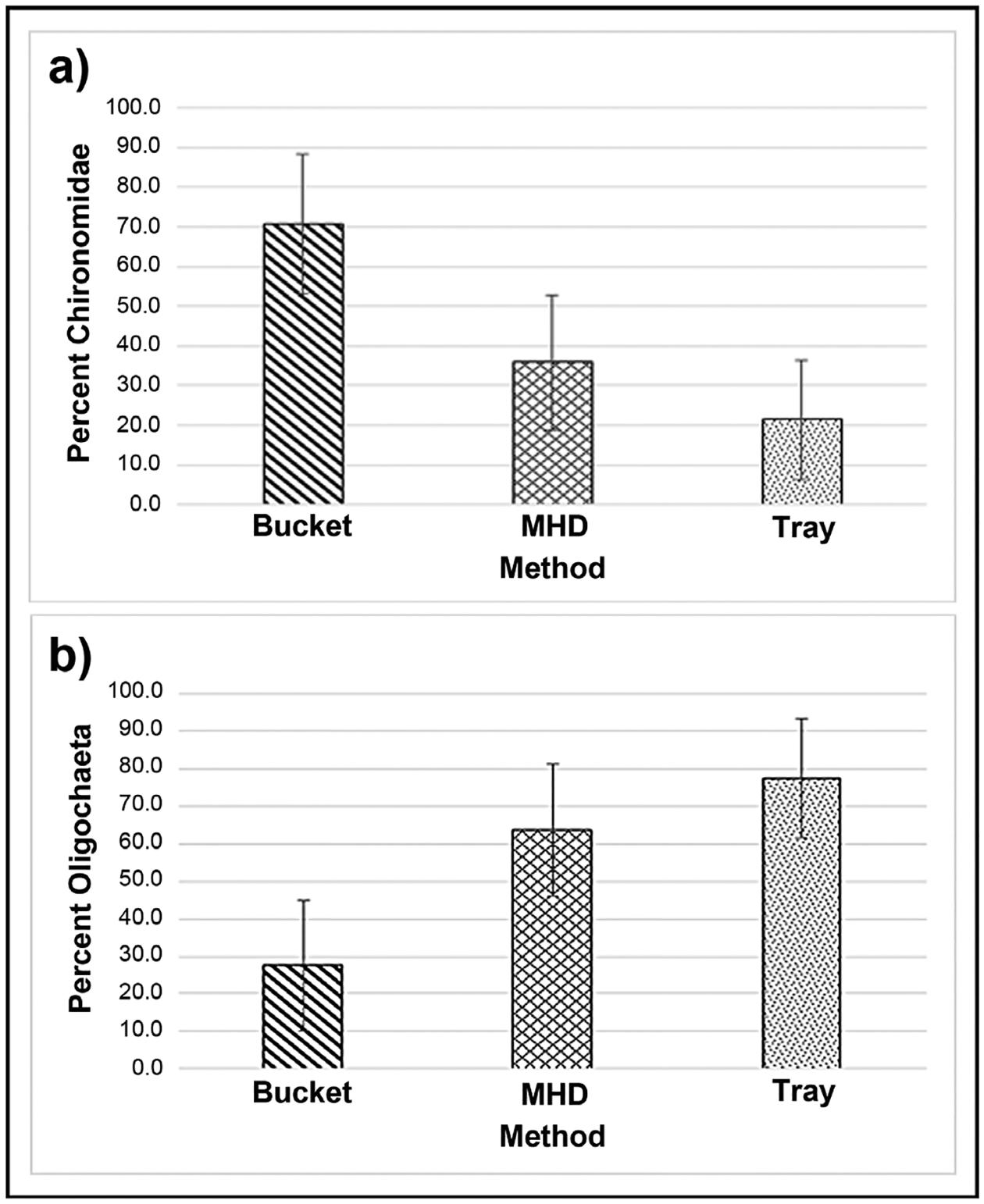
Average percentage ± 1 SD of (a) chironomid abundance and (b) oligochaete abundance per sample.

The dominant taxonomic groups in all samples were Chironomidae and Oligochaeta; however, the sample makeup of these groups varied with method (Figure 9). With few exceptions, Chironomidae were the dominant taxonomic group retrieved with the bucket grab sampler while Oligochaeta were dominant for the trays and MHD samplers. The communities from the bucket grab samples had a significantly higher mean percentage (71%) of chironomids (Figure 9(a)) than those from the MHDs (36%, p = 0.002) or trays (28%, p < 0.001). The communities from the bucket grab samples had a significantly lower mean percentage (28%) of oligochaetes (Figure 9(b)) than those from the MHDs (63%, p = 0.002) or trays (77%, p < 0.001).

**Figure 10.**
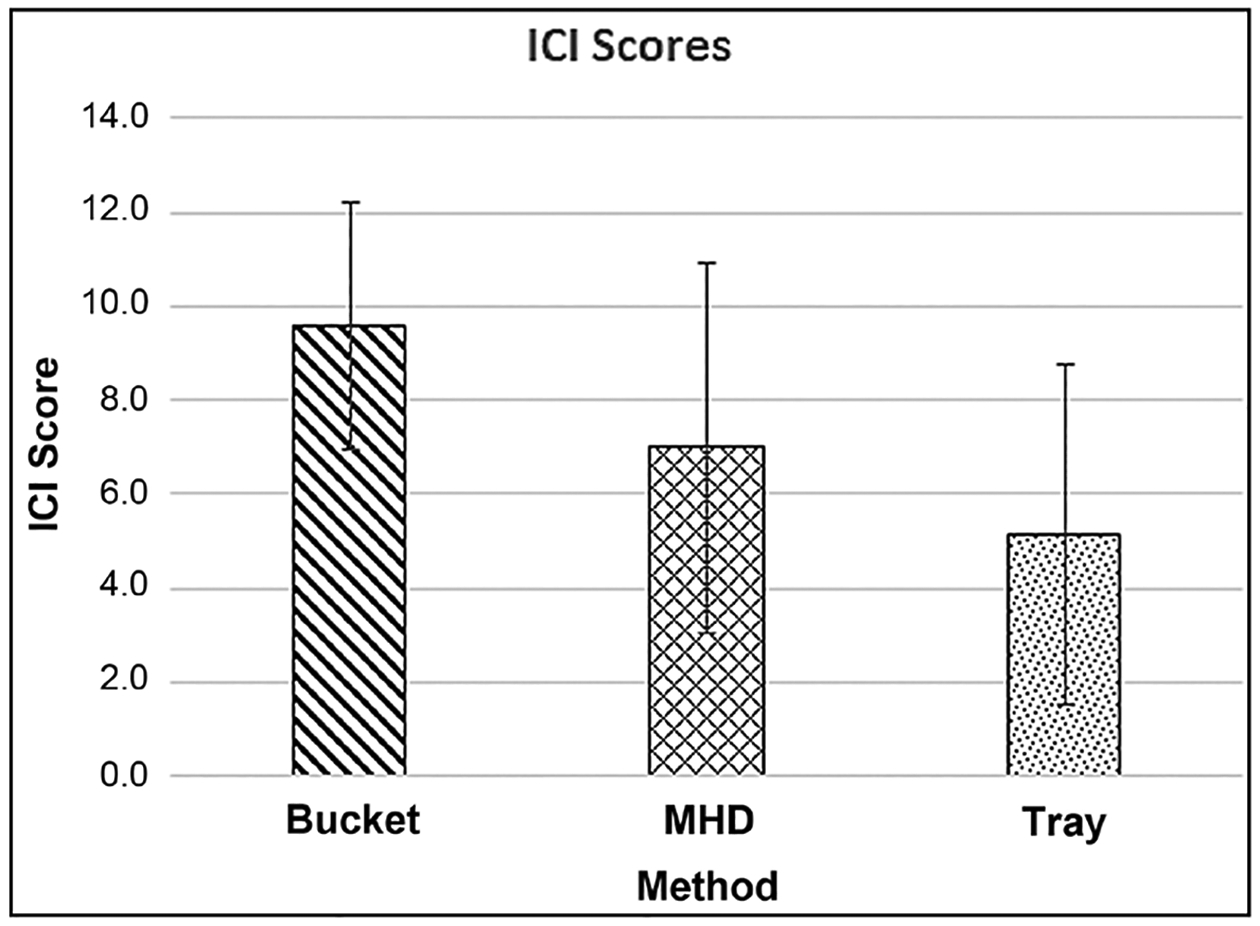
Comparison of mean ICI scores ± 1 SD calculated by the 3 methods.

Figure 10 shows the mean Invertebrate Community Index (ICI) scores [17] calculated from the macroinvertebrates retrieved by each method. There were no statistically significant differences in ICI scores, between methods. Based on these ICI scores, 15 of the 23 samples (all methods) resulted in a water quality rating of “Poor”, and 8 samples had a rating of “Very Poor”, with an overall average rating for this section of Congress Run (pooling upstream and downstream pool samples) of “Poor”. The upstream pool had an overall rating (all methods) of “Very Poor”, and the downstream pool had an overall rating (all methods) of “Poor”. The rating of this section of the stream (pooling upstream and downstream pool samples), varied by method. Averaging all the bucket replicate samples into one rating resulted in a water quality rating of “Poor”. Doing the same for the MHD or tray replicates resulted in a “Very Poor” rating. Each method had samples with a “Very Poor” rating. All of the samples with “Very Poor” ratings were from the pool above the culvert. Other than total taxa, which is one metric of the ICI, other ICI metrics that responded differently among methods were the total number of dipteran taxa and percent tolerant organisms. For the total number of dipteran taxa metric, an ANOVA indicated that the bucket method had a greater mean number of taxa (13.9) than the tray method (mean = 5.7, p < 0.001) and the MHD method (mean = 8.2, p = 0.009). For the percent tolerant organisms metric, the bucket method had a lower percentage of tolerant taxa (56.0%) than the tray method (mean = 82.1%, p = 0.028), but not the MHD method (mean = 75.6%, p = 0.067). The percent tribe tanytarsini, and other diptera and non-insects metrics showed no difference between methods. For the five metrics that involved the EPT taxa there were of course no differences seen between methods as there were no EPT taxa retrieved from the pools in this particular sampling.

**Figure 11.**
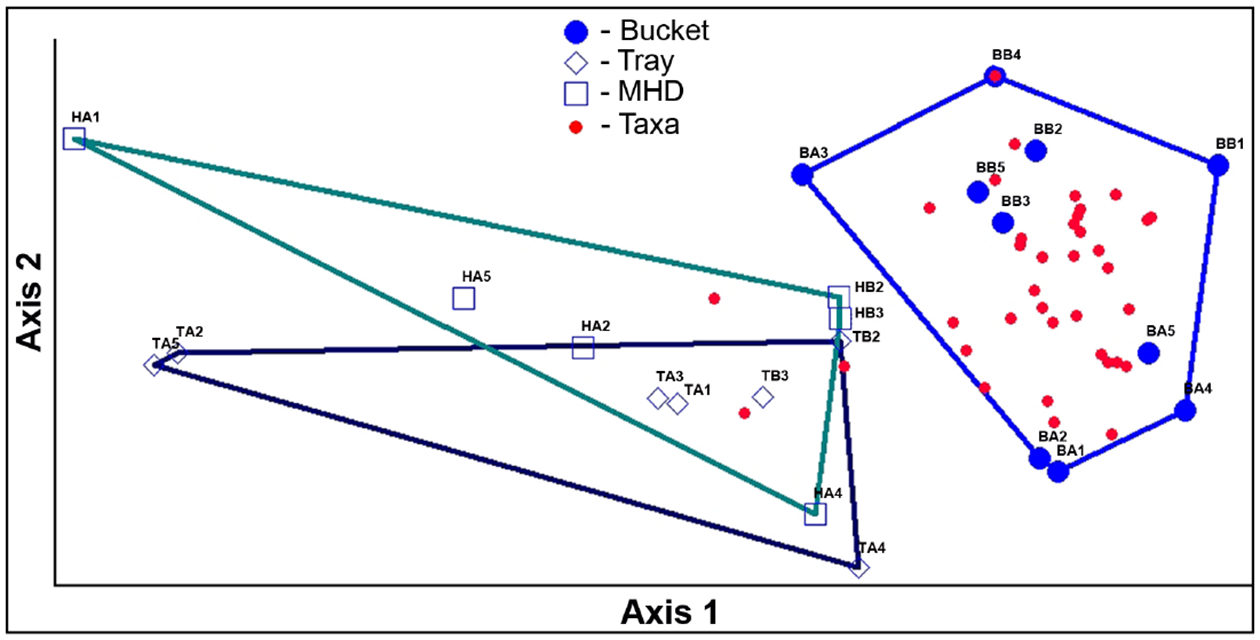
Grouping of macroinvertebrate assemblages by method. Results of nonmetric multidimensional scaling (NMDS) analysis of the samples plotted in taxa space showing grouping and similarity of the assemblages of the bucket method samples. [Sample codes: First letter indicates the method (B-bucket, T = tray, H = MHD); second letter indicates location (A = above culvert, B = below culvert); number indicates the replicate.]

**Figure 12.**
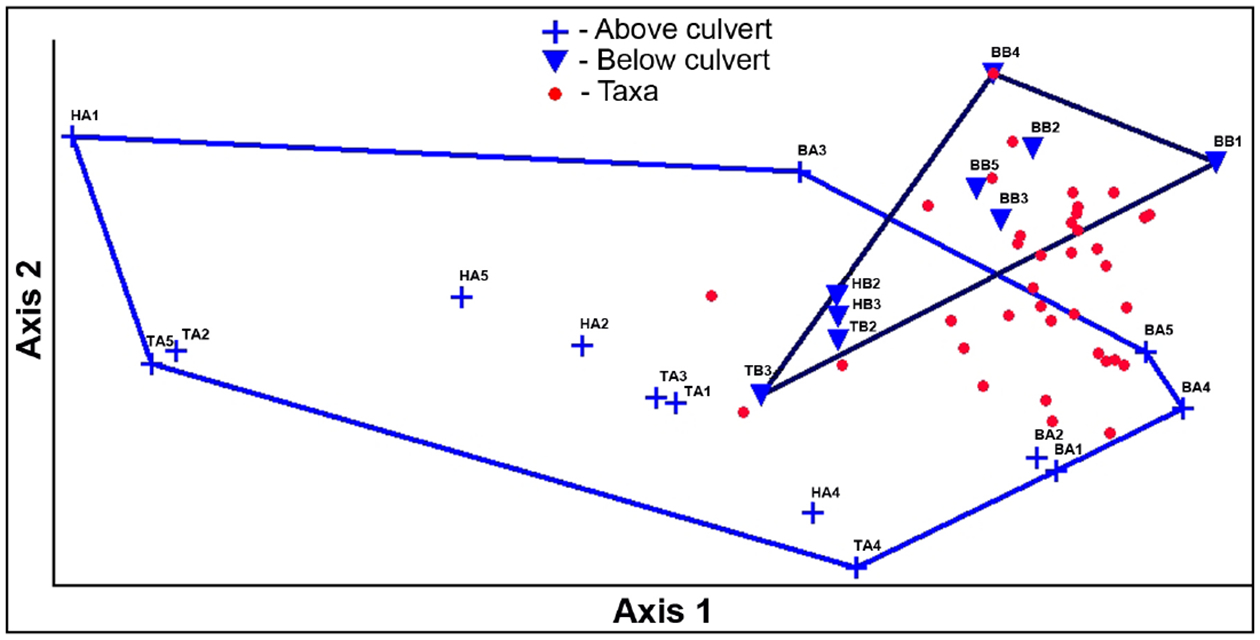
Grouping of macroinvertebrate assemblages by location. Results of nonmetric multidimensional scaling (NMDS) analysis of the samples plotted in taxa space showing grouping. [Sample codes: First letter indicates the method (B-bucket, T = tray, H = MHD); second letter indicates location (A = above culvert, B = below culvert); number indicates the replicate.]

Multivariate analyses (NMDS and MRPP) revealed some significant differences in the macroinvertebrate assemblages when grouped by method (Figure 11), or by location (Figure 12). The NMDS analysis showed a good fit, with a stress of 4.93.

The graphic representation of the NMDS in Figure 12, shows the grouping of macroinvertebrate assemblages retrieved by the bucket grab samples, and differences between these assemblages and those retrieved from the deployed samplers. There was also some grouping seen based on location, whether the samples were from the above or below the culvert (Figure 12).

MRPP analyses give statistical confirmation of the groupings seen visually in Figure 11 and Figure 12. An MRPP analysis run on the groups of samples representing the three methods (Figure 11) gives the following values: T = −6.15, p = 0.0002, and A = 0.276. When methods are grouped by bucket or deployed sampler (HD + tray), a larger difference between methods was seen, with a more negative T (−8.72), smaller p (0.00007), and a similar A (0.269) value. An MRPP analysis run on the groups of samples located above and below the culvert (NMDS shown in Figure 12) indicates significant differences in the communities: T = −6.48, p = 0.00061, and A = 0.200.

## 4. Discussion

This study shows once again the importance of researching the best sampling method to meet specific waterbody conditions and study objectives. Hester-Dendy and rock basket/tray methods are widely used and useful in many situations. The effectiveness of gravel-filled colonization trays for characterizing the macroinvertebrate community has been demonstrated in previous studies [37] [38] [39]. Hester-Dendy samplers have been used with success by federal [14] and state [7] [15] [16] [18] [40] agencies. However, neither of these samplers, even when modified for this situation (low-profile MHD) represented the best method for this flashy urban stream. For streams with diverse communities, identification of macroinvertebrates to family level can be sufficient to detect spatial or temporal differences in water quality [41] [42]. However, for highly impacted streams like Congress Run, composed mostly of Chironomidae and Oligochaeta, identification of Chironomidae to lowest achievable level (usually genus or species), along with a focus on chironomid community-based metrics [43] [44], and multivariate statistical analyses like NMDS is likely necessary to detect these differences. Future analyses of water quality would warrant incorporation of analyses of chironomid traits and life history strategies [45] as another means of detecting changes in the level of anthropogenic disturbance.

The ICI is a well-established index, relevant to Ohio Streams, and includes Chironomidae metrics, thus useful for bioassessment of Congress Run. However, the ICI values and ratings calculated in this study should not be considered completely comparable to Ohio EPA ICI ratings, as our methods did not adhere to the Ohio EPA sampling protocols [15] [16] [17]. Deviances from the Ohio EPA method include that our MHDs were modified from the Ohio EPA design, we did not adhere to the recommended retrieval period of June 15 to September 30, and the flow at our sites did not meet the ≥10 cm/sec level that the Ohio EPA recommends for accurate interpretation of the ICI.

Though no EPT taxa were found in these spring sampling data, Ephemeroptera (Heptageniidae, Caenidae) were found in small numbers in other grab-samples. This available pool of taxa, along with additional taxa found in nearby Mill Creek (including a number of Trichoptera and Ephemeroptera taxa), indicates a potential for recovery of the ICI and water quality if enough green infrastructure and remediation efforts were undertaken within the watershed to significantly alter the flashy hydrology. Future research needs include collecting in the fall season, as well as exploring ways to further standardize the bucket method to reduce variability.

### 4.1. Usability

The bucket grab sampler required the least effort to sample, process, sort, and ID. The shorter time needed for sorting of the bucket samples makes sense in light of the lesser amount of sediment collected in the bucket samples compared to samples from the deployed samplers, and the lower abundance of the deployed samplers (especially the tray samplers). Therefore, a larger volume of sediment had to be picked through (usually representing the entire sampler) for the deployed samplers in an attempt to get to a minimum subsample of 100 organisms. The bucket sampler was the only one which resulted in a complete set of 5 samples from each sampling site.

The MHDs and trays were candidates for use in urban streams, in large part because they don’t require flow to collect a sample, like many commonly used samplers (Hess, Surber, kick net) and thus could be used in the isolated pools found for a large part of the year in flashy urban streams. However, this advantage was largely counteracted by being buried and swept away by periodic spates. Since the USGS gage on Congress Run is new, there is insufficient historical data to compare stream surges on Congress Run during our deployment period to stream surges that might bury or sweep away deployed samplers over multiple years. However, there is a nearby gage on Mill Creek (USGS 03259000 Mill Creek at Carthage OH) in the same watershed that can be used to compare multiple years of stream hydrology. In 5 of the 7 years from 2012–2018, 2 to 3 m surges occurred during our spring deployment period (April 26 through June 8) at this nearby gage on Mill Creek. In 2012 and 2017 there was a surge of the same height (~3½ m) at this Mill Creek gage, that corresponded to the surge seen on Congress Run April 28–29 in 2017 (Figure 5).

An attempt was made to include another stream in this methods study; one expected to be less affected by urban disturbances, the West Fork of the Mill Creek. The West Fork runs through a forested city park and is of similar size to Congress Run, but its watershed has less industrialization and urbanization. This stream turned out to have even more powerful surges from rain events (perhaps due to a steeper slope), and all of the MHD and tray samplers were washed away. In addition, we had issues with vandalism. For these reasons, the bucket grab sampler was the only one of our 3 methods suitable for this location.

Other researchers have also experienced some of the usability issues of artificial samplers associated with high flow that we found in this study. Roby *et al.* [46] found that artificial samplers “were lost, became clogged or buried”. Kirk and Perry [47] reported that multi-plate samplers were washed downriver at high flows. Monitoring programs have tried to account for flow issues by incorporating different means of anchoring the samplers. Though attaching the deployed samplers to rebar driven into the sediment (in addition to the attached weights) may help somewhat in keeping the samplers in place during spates, we had mixed success with attempts to do this, and this added measure did nothing to keep the samplers from being buried or to ensure that the samplers will remain within the flowing portion of the channel. The state of Iowa [18] uses a type of Hester-Dendy sampler which a modified for low flow conditions, with rods holding the samplers above the surface of the sediment. This design can apparently help with the issue of burial, but the IDNR methods also acknowledge the possibility of damage from high flows.

### 4.2. Variability

The variability for all of these methods was higher than desired for a number of metrics. However, for total taxa richness, the most commonly used metric by U.S. state agencies [8], the bucket method performed well with respect to variability (CV of 20.9%). It also had the lowest CV for the ICI multi-metric index (Table 1), giving this method greater ability to detect differences in water quality between site samplings. The abundance values from the bucket grab samplers varied greatly (Figure 8) but, due to higher abundances, did not have a higher CV. Though the variability associated with the tray and MHD samplers was higher than that of the bucket grab samplers for this study, one would not expect that result to necessarily extend to their use in non-flashy streams. The results from previous studies of comparisons of the variability of grab samplers versus deployed artificial substrate samplers are mixed. Some studies have found that artificial substrate samplers had lower variability [38] [39] [46] [48] while others have found grab samplers to have a lower variability [49] [50]. Based on long-term use, Hester-Dendy multi-plate samplers have shown a variability which is acceptable for use for monitoring and assessing water quality in lotic and lentic waterbodies by state programs including Ohio [15] [16] and New York [40], and by the U.S. EPA [14]. However, these programs utilize modified Hester-Dendy samplers and to a lesser extent rock-filled trays, primarily to monitor waterbodies with a greater hydrological permanence (rivers, perennial streams, lakes) than exhibited by flashy urban streams. The MHD and tray samplers ranged from being partly to completely buried. Some of these replicates that were buried the deepest had very low abundances (<20 individuals), which likely contributed to the higher variability of MHDs and trays.

### 4.3. Macroinvertebrate Community Retrieval Completeness

Multiple factors may potentially contribute to the differences in assemblages seen between methods, including the greater abundance and diversity seen in the communities retrieved by the bucket method. First, the significantly greater area/footprint of the bucket sampler should be considered a prime factor. The cross-sectional area of the MHD is approximately half that of the cross-sectional area of the trays and about 20% of the cross-sectional area of the bucket. Though the additional scraped rocks would add to the area of the bucket method, qualitative observations during sampling were of no noticeable macroinvertebrate presence on the rocks. Also, the macroinvertebrate taxa we had hoped to find from this extra sampling activity, which might utilize the surfaces of the rocks (Trichoptera, Ephemeroptera), were not found in any of the bucket samples. Researchers interested in using the bucket method, for whom it is more important to be able to calculate an accurate area for density calculations than maximizing the number of taxa retrieved, or where there is no noticeable macroinvertebrate presence on rocks in the sampling area, may not want to include these extra rocks as part of the sampling method.

Another potential factor affecting differences in communities is differences in particle size distribution between methods. Several studies [51] [52] [53] [54] [55] have found an effect of substrate particle size on the makeup of benthic macroinvertebrate assemblages, with different taxa having preferences for different particle sizes. One quantified particle size difference in this study was that of the MHDs having less of the medium to coarse gravel-sized particles (>5.6 mm) than the other methods. This makes sense due to the small openings (2 – 3 mm) between plates in these samplers, which would represent an obstruction to larger sized particles. In addition, though the silt portion of sediments was not collected and quantified during sample processing, observations during sampling and sample processing were of the substrate in pools where the bucket samples were taken having a higher proportion of silt than was present in the MHD and tray samples.

The deployed samplers being buried in deposited sediment early in the sampling period represent another potential factor affecting differences in macroinvertebrate assemblages between methods. Artificial substrate samplers like the gravel tray and MHD, can provide macroinvertebrate habitat that is not abundant in pools like the ones in Congress Run, which have a substrate of mostly sand and small to medium gravel. This introduced habitat, including hard surfaces and spaces for attachment and refuge, brings with it the potential to retrieve some additional taxa from these pools. However, this potential is lost when these samplers are buried in sediment when spates and the associated sediment deposition occur. Since the sediment on top of the gravel tray and MHD samplers is not collected and analyzed, the habitat assessed in these deployed samples is different from that assessed by the bucket sampler. In the bucket method, approximately the top 10 cm of sediment is stirred up and sampled, so macroinvertebrates are retrieved from a range of depths, from the sediment surface down to 10 cm. Therefore, the tray and MHD samplers, whose surfaces were several cm below the surface by time of retrieval, would generally be collecting macroinvertebrates from a narrower, deeper sediment habitat than the bucket grab samples. The differences in habitat sampled by the different methods likely contribute to the differences in the assemblages retrieved by the 3 sampling methods. These differences in habitat sampled could help to explain why Chironomidae were the dominant taxonomic group retrieved by the bucket grab samples, while Oligochaeta were dominant in the assemblages from the deployed samplers (Figure 9). Taxa may have preferences regarding the depth in the sediment at which they reside. For example, Williams and Hynes [56] recovered Oligochaeta from significantly deeper levels of the sediments than Chironomidae. It is quite possible that higher percentages of Chironomidae in the assemblages retrieved from the bucket samplers contributed to the higher chironomid and total taxa richness for the bucket grab sampler versus the deployed methods.

Due to these multiple factors that could potentially affect the differences in assemblages among sampling methods, we did not attempt a quantitative normalization by area among methods nor focus on analysis of method differences based on a comparison of densities. This follows the example of researchers in previous studies that compared artificial substrate samplers (rock baskets, multi-plate, or webbing) and grab samplers (fixed area (e.g., Surber) and/or dredge (e.g., Ponar) and did not attempt such a normalization [46] [49] [55] [57] [58] [59]. Only a few U.S. State agencies use density as a metric, while measures of richness are by far the most popular type of metric used by these agencies [8]. Therefore, for most studies, which method will more completely retrieve the number of taxa living in the stream will likely be a key consideration in the decision in the choice of sampling method.

The bucket grab sampling method performed best with respect to our criteria of usability, variability, and community retrieval, and will be used going forward to assess the green infrastructure/restoration effort in Congress Run. The bucket grab sampler has the potential to help fill the methods gap that exists for quantitative sampling of macroinvertebrates in flashy urban streams, whose flow regimes pose problems for commonly used grab and deployed sampling methods.

## Figures and Tables

**Table 1.** Coefficients of Variation (CV) (percent) for the 3 sampling methods and all deployed samplers (MHD + tray) for some common metrics.

		# Taxa	# Chironomid Taxa	# Non-chironomid Taxa	Abundance	Abundance Chironomids	Abundance Oligochaetes	ICI Rating
**Method**	Bucket	20.9	23.5	35.6	54.6	65.3	100.4	27.4
	MHD	42.9	41.4	62.0	80.5	117.7	81.8	56.4
	Tray	53.9	55.5	57.7	77.6	140.4	85.4	70.5
	Deployed	48.4	48.5	59.9	79.0	129.1	83.6	62.4

**Table 2.** List of taxa retrieved by all methods (bucket + MHD + tray) in the 2017 spring sampling of the section of Congress Run upstream and downstream of the culvert, just above the confluence of Congress Run with Mill Creek.

Chironomid Taxa	Non-Chironomid Taxa
*Ablabesmyia* sp.	Oligochaeta
*Alotanypus* sp.	Collembola
*Zavrelimyia sp*.	Undetermined Diptera
*Psectrotanypus*	Ephydridae
*Prodadius*	*Atrichopogon* sp.
*Conchapelopia* sp.	Culicidae
*Corynoneura* sp.	*Undetermined Hirudinea*
*Cricotopus bicinctus*	Nematoda
*Cricotopus/ Orthocladius* gr.	Isopoda/ Caecidotea sp.
*Thienemanniella xena*	Gastropoda
*Eukiefferiella claripennis*	Ancylidae
*Chiron om us* sp.	Lymnaeidae
*Cryptochironomus*	Physidae
*Dicrotendipes* sp.	Pelecypoda
*Dicrotendipes simpsoni*	Elmidae
*Microtendipes* sp.	Turbellaria
*Paratendipes* sp.	*Hydra* sp./Hydroida
*Phaenopsectra punctipes*	
*Phaenopsectra obediens*	
*Polypedilum idinoense* gr.	
*Polypedilum fallax*	
*Polypedilum scalaenum* gr.	
*Micropsectra* sp.	
*Paratanytarsus* sp.	
*Rheotanytarsus* sp.	
*Tanytarsus sp*. (Prob. *guerlus*)	
